# Iatrogenic Neonatal Esophageal Perforation: A European Multicentre Review on Management and Outcomes

**DOI:** 10.3390/children10020217

**Published:** 2023-01-26

**Authors:** Eva Sorensen, Connie Yu, Shu-Ling Chuang, Paola Midrio, Leopoldo Martinez, Mathew Nash, Ingo Jester, Amulya K. Saxena

**Affiliations:** 1 Chelsea Children’s Hospital, Chelsea and Westminster Hospital NHS Foundation Trust, Imperial College London, London SW10 9NH, UK; 2 Department of Pediatric Surgery, Cà Foncello Regional Hospital, 31100 Treviso, Italy; 3 Department of Pediatric Surgery, University Hospital La Paz, 28046 Madrid, Spain; 4 Birmingham Children’s Hospital, Birmingham Women’s and Children’s NHS Foundation Trust, Birmingham B15 2TG, UK

**Keywords:** esophageal perforation, neonates, nasogastric tube, management, outcomes

## Abstract

Background: The aim of this multicenter retrospective study and literature review was to review management and outcomes of neonatal esophageal perforation (NEP). Methods: Protocol data were collected from four European Centers on gestational age, factors surrounding feeding tube insertion, management and outcomes. Results: The 5-year study period (2014–2018) identified eight neonates with median gestational age of 26 + 4 weeks (23 + 4–39) and median birth weight 636 g (511–3500). All patients had NEP from enterogastric tube insertions, with the perforation occurring at median 1st day of life (range 0–25). Seven/eight patients were ventilated (two/seven-high frequency oscillation). NEP became apparent on first tube placement (*n* = 1), first change (*n* = 5), and after multiple changes (*n* = 2). Site of perforation was known in six (distal *n* = 3, proximal *n* = 2 and middle *n* = 1). Diagnosis was established by respiratory distress (*n* = 4), respiratory distress and sepsis (*n* = 2) and post-insertion chest X-ray (*n* = 2). Management in all patients included antibiotics and parenteral nutrition with two/eight receiving steroids and ranitidine, one/eight steroids only and one/eight ranitidine only. One neonate had a gastrostomy inserted, while in another an enterogastric tube was orally successfully re-inserted. Two neonates developed pleural effusion and/or mediastinal abscess requiring chest tube. Three neonates had significant morbidities (related to prematurity) and there was one death 10 days post-perforation (related to prematurity complications). Conclusions: NEP during NGT insertion is rare even in premature infants after evaluating data from four tertiary centers and reviewing the literature. In this small cohort, conservative management seems to be safe. A larger sample size will be necessary to answer questions on efficacy of antibiotics, antacids and NGT re-insertion time frame in NEP.

## 1. Introduction

Orogastric or nasogastric tube (‘enterogastric tube’) insertion is a common procedure undertaken in the neonatal unit. Neonatal esophageal perforation (NEP) is a known, but rare, complication of enterogastric tube insertion in pre-term and low birth weight infants [1,2]. In Singh et al.’s [3] review of neonates with a birth weight of under 500 grams over a 10-year period, there were 3 NEPs related to nasogastric tube in their very low birth-weight cohort of 28. Filippi et al. [2] calculated an incidence of NEP of 1 in 124 in very low birth weight neonates, which increased to 1 in 25 for those born weighing less than 750 g. Thanhaeuser et al. [4] calculated an incidence of upper gastrointestinal tract perforations of 1.1% in their extremely low birth weight infants, of which less than half were esophageal perforations. The most common causes of esophageal perforation in neonates are endotracheal intubation, airway/upper gastrointestinal suction and enterogastric intubation [5,6,7]. However, there are also reports of perforation secondary to transesophageal probe placement [8,9]. NEP is often asymptomatic and only identified through radiological imaging [6], but symptoms such as respiratory distress, cyanosis, choking, vomiting [10] or evidence of sepsis [1] can develop. NEP is associated with a mortality rate of up to 30% [11,12] though this is usually through complications of prematurity rather than as a direct result of the iatrogenic injury [11].

The management of esophageal injuries in adults often involves operative drainage, repair and diversion [13,14]. In recent years there has been a shift from surgical to non-operative management for isolated esophageal injuries in the neonatal population [15,16], and in the absence of congenital abnormalities of the esophagus such as esophageal atresia [14]. However, the specifics of non-operative management are not well defined, with a paucity of published evidence regarding several aspects of this management. The aim of this study, therefore, was to describe the management and outcomes of this rare complication in four neonatal surgical centers across Europe. 

## 2. Methods

This was a retrospective cohort study of neonates admitted to four European neonatal surgical centers (Chelsea and Westminster Hospital NHS Foundation Trust, London, UK, Cà Foncello Regional Hospital, Treviso, Italy, University Hospital La Paz, Madrid, Spain and, Birmingham Women’s and Children’s NHS Foundation Trust, Birmingham, UK) from 2014 to 2018. Data were collected through retrospective chart review on a standardized protocol form Supplementary Materials File S1. Data recorded included basic demographics, associated anomalies, and the cardiorespiratory status of the infant prior to the perforation. Furthermore, the circumstances surrounding the enterogastric tube insertion were recorded, alongside the discovery and diagnosis of the perforation (including any clinical signs that raised suspicion and the use of radiographs). Several aspects of medical and surgical management were also recorded. Morbidity and mortality data for minimum of two years post-injury was obtained. The study was registered locally as a service evaluation at each center. 

All neonates (under 44 weeks corrected gestational age) with a diagnosis of NEP were eligible for inclusion. Those with a diagnosis of esophageal atresia and/or trachea-esophageal fistula were excluded. Primary end points were to determine the sample size, presenting symptoms, diagnosis, management and mortality. Secondary outcomes were age and weight correlation, primary insertion or tube change and ventilation parameters.

Descriptive statistics were used for analysis using Microsoft Office Excel for Mac (Version 16.69, Microsoft, Redmond, WA, USA). Data is presented as median (IQR), or N (%). 

## 3. Results

Eight patients with an isolated NEP were identified across the four centers during the study period (Table 1). The median gestational age at birth was 26 + 4 weeks (24 + 4–30 weeks). Only one (12.5%) patient was born at term (>37 weeks). The median birth weight was 636 g (563.8–1109.3 g) with six patients (75%) being extremely low birth weight.

There were no significant underlying comorbidities, other than prematurity and associated conditions, such as intraventricular hemorrhage.

The diagnosis of NEP occurred on median day 2 of life (0–11). Seven patients (87.5%) were intubated and ventilated at the time of diagnosis, of which five were on conventional ventilation and two undergoing high frequency oscillation. One patient was self-ventilating on air. 

In all eight patients, an enterogastric tube was placed immediately after birth. Perforation became apparent most commonly at the first tube change (5, 62.5%), after multiple tube changes (2, 25%), and on one occasion, on first tube placement (12.5%).

The diagnosis was established during enterogastric tube insertion for one patient (12.5%), as they developed respiratory distress immediately. Two patients (25%) were asymptomatic of perforation but the diagnosis became apparent on the post-insertion chest X-ray. For the remaining patients, NEP was suspected due to respiratory distress alone (3, 37.5%) or in conjunction with signs of sepsis or shock (2, 25%). 

The site of esophageal perforation was known in six patients. Of these, three patients perforated at the distal esophagus (50%), whilst two (33%) patients had a proximal esophageal perforation and one (17%) had a mid-esophageal perforation. 

All eight patients had their enterogastric tube removed, were made nil by mouth, and total parenteral nutrition (TPN) and intravenous antibiotics started. Other medications included steroids (3, 37.5%), and two patients (25%) received intravenous ranitidine. Antibiotic regime varied between centers. The median duration of antibiotics was 10 days (7–35). Oral feeds were started after a media of ten days (10–37). Parenteral nutrition was continued until full enteral feeds established in all patients. 

Two patients required a chest drain insertion: one due to pneumothorax with pleural effusion and the other due to mediastinal abscess and pleural effusion. Their chest drains remained in place for 12 and 34 days, respectively. Both patients were offered gastrostomies. One patient underwent gastrostomy insertion on day 16 post-injury. The other had a successful orogastric tube insertion under fluoroscopic guidance on the operating table and therefore gastrostomy was not performed. Importantly, no other surgical procedures, such as primary repair of the perforation, was offered to any patient. 

There was one mortality within the cohort, 10 days after the NEP. The cause of death was severe respiratory distress syndrome with acute on chronic renal failure. Three patients were diagnosed with chronic lung disease. No patients suffered from esophageal stenosis or stricture.

## 4. Discussion

Esophageal perforation in neonates and premature infants is unusual and the number of cases is limited. These perforations are generally iatrogenic and three major interventions could be implicated in their occurrence: multiple attempts at intubation, insertion of an orogastric or nasogastric tube and suction of the pharynx. None of these interventions are avoidable in neonates who have to undergo them; however, multiple attempts at intubation could be sidelined as the single event among these where the neonate is extremely ill and emergency intubation is a lifesaving intervention. These interventions are performed by experienced doctors that are trained in neonatal intensive care; however, the size of the neonate, the emergency situation and possible difficult anatomy may be implicated broadly as contributing factors. Moving on to the insertion of a nasogastric or orogastric tube, this could be considered both an emergency at the time of birth or a standard procedure performed by both doctors and nurses in premature and term neonates. The procedure of inserting the orogastric or nasogastric tube beyond the orifice is based on human skill factors and cannot be controlled or monitored until the tube has been positioned in its final resting place, following which an abdominal film is taken to confirm its position. Ideally, it would be desirable to follow the pathway of the tube as it passes beyond the oropharynx; however, this would be an impossible task in any setting. The low incidence of esophageal perforations has suggested that this prevalent procedure is safe in all neonatal age groups. The change in these tubes is mandated if these tubes are blocked, dislocated or associated with pressure-related problems at the point of insertion, as this may affect the administration of feeds. These changes are performed by experienced nursing staff of neonatal intensive care units. There are however individual and varying protocols at intensive care stations worldwide regarding their regular change; a non-emergency factor that has been implicated in esophageal perforations. It is worth noting that both professional groups, doctors and nurses are involved in procedures that could contribute to neonatal esophageal perforations.

With the rise in the number of premature infants in intensive care units across the globe, it would be presumed that this would be a more common occurrence; however, our report also indicates that the number of these cases in this subpopulation is extremely low and patients need to be pooled to obtain numbers to understand outcomes. It is obvious that although major tertiary centers in Europe have contributed their cases, the sample size is low and the calculation of outcomes is difficult. It is also difficult to estimate the numbers throughout Europe as reporting on individual cases do not present novel publication. Even if such numbers are pooled, it is challenging to separate neonatal intensive care stations regarding their case volumes bearing in mind that this could be a contributing factor when low-volume centers are challenged with extremely premature low birth weight neonates. Additionally, due to low reporting, there is no obvious trend as to whether cases of neonatal esophageal perforations were higher in the past decades or are higher during contemporary times where the trend to manage extremely premature infants presenting with complex conditions has become more common. As there are no benchmarks, data collection from national cohorts will need to be collected to estimate incidences and analyzed to compare the outcomes and approaches in low-volume versus high-volume centers. Multiple variables such as the age and weight of the neonate, comorbidities, coexisting malformations or patho-morphological conditions in the oropharynx or chest, diagnostic and management approach will need to be combined to give a better understanding of outcomes.

In our small cohort of patients, NEP was only observed in low birth-weight and/or premature infants. Some patients had evidence of deterioration, but overall the diagnosis was radiographic. Our patients were successfully managed conservatively, with the only surgical intervention being gastrostomy insertion. Non-surgical management included antibiotics, a period of nil by mouth, and TPN; with chest drain insertion if the clinical condition dictated it. In the minimum-two-year follow up period, no complications of the NEP were noted. 

In our cohort, radiological findings alongside respiratory deterioration indicated the diagnosis of NEP. This echoes the literature where routine post-tube insertion X-ray [5,7], and acute deterioration in respiratory status [6,10,17,18] led to the diagnosis of NEP. X-ray may show an abnormal path of the enterogastric tube [7,19], or pneumothorax/pneumomediastinum with or without pleural effusion [1,5,6,10]. The diagnosis may also be considered if the gastric aspirates are blood-stained immediately after tube insertion [20]. It is worth noting that absence of radiological evidence of a perforated esophagus does not necessarily rule it out [20]. Failure to establish the diagnosis prior to commencing feeds can have tragic outcomes [21]. Soong (26) reports on three cases where flexible endoscopy was used to diagnose esophageal injury and precisely identify the location of the perforation. More commonly, esophagogram confirms a leak [7,9,22], or is used to rule out persistent leak prior to commencing oral feeds [5,9,22,23]. 

The mechanism of neonatal esophageal perforation needs to be revisited. Materials that constitute enterogastric tubes are generally polyvinyl chloride (PVC), polyurethane (PUR), and silicone. PVC is a non-tissue compatible material and it has the tendency to become brittle and hence is a short-term tube <7 days. PUR is tissue compatible and the material of choice in fine bore nasogastric tubes as it remains soft and flexible throughout use. Silicone is tissue compatible however its flexible state makes it unsuitable for neonatal applications. The limitation of this study has been that the materials that caused the perforations were not investigated. Additionally, the lubrication technique of the tubes before insertion which could be important factor was not investigated. However, it must be noted that this data may be difficult to collect retrospectively.

The stiffness of the tube of small size almost acts as a needle when advanced through the esophagus of a neonate. The immature esophagus wall offers minimal resistance compared to that of infants, children or adults during the passage of the tube through the esophagus. The lack of noticeable resistance during the passage of these tubes in premature and term neonates is a significant factor in these perforations going unnoticed at the time of tube placements that have caused perforations late presentation of symptoms such as bleeding through the tube, suction of blood-tinged saliva in the oropharynx and deterioration in the condition of the neonate are often the first signs with the suspicion of such injuries. If the nasogastric or orogastric tube is used for gastric decompression the incidence may go unnoticed for some time, when compared to those tubes that are used for feeding which introduce feeds either into the mediastinum, chest or abdominal cavity and trigger sudden deterioration in the condition of the neonate. Serial chest or abdominal films are generally not part of the protocol to control the position of nasogastric or orogastric tubes in neonates. However, even if films are performed for other reasons, it is often that these perforations may be difficult to pick up on native films.

Half of our patients, where the site of perforation was known, suffered a perforation in the distal esophagus. Wolf et al. [24] describe three types of injury in their case series from enterogastric tube insertion: posterior pharyngeal rupture, non-complicated esophageal rupture with formation of a false lumen or complicated esophageal rupture with penetration into the right pleural space. Although the usual route of perforation is into mediastinal, pleural and peritoneal cavities, there are reports from less recent literature of the tube entering other organs, including the pericardial sac [25], the renal pelvis [26] and the urinary bladder [27]. 

The dilemmas of approach from the pediatric surgeons are multifold as the decision to intervene surgically is extremely challenging in sick and extremely premature infants. It is often difficult to localize the location of the perforation based on native films and contrast dyes are often utilized as a tool to confirm the diagnosis and the extent of the problem. In perforation restricted to the mediastinum, interventions are not necessary. However, if there is a breach of the pleura along with accumulation of feeds in the hemi-thorax, depending on the amount a decision may be taken to perform a one-off pleurocentesis or to place a chest tube. Tears of the esophagus are generally rare with orogastric or nasogastric tubes, however these are more associated with multiple intubation events in which a larger bore endotracheal tube is pushed through the esophagus. These tears would generally involve the proximal esophagus. The stiffness of the endotracheal tube as well as the curved structure are additional factors that attribute to esophageal tears. Limiting factors in surgical intervention in such tears is the involvement of proximal esophagus close to the thoracic inlet, the difficulties in optimal surgical access of this area and the morbidity or lethal outcome in an extreme premature neonate. The possibility of the tube ending into the abdomen after para esophageal passage through the diaphragm and feed introduction into the abdomen would require paracentesis or placement of a drain to evacuate large accumulations.

Gaining access to the stomach for establishment of feeds is another issue after the diagnosis of esophageal perforations in neonates. Reinsertion of the orogastric or nasogastric tube is fraught with the danger of the tube passing again through the perforation taking the same trajectory as the tube that caused the injury or causing a tear in the perforation as the tissue could be inflamed after the perforation. The options that are preferred would be to maintain the neonate with total parenteral nutrition if there is adequate vascular access or to perform a gastrostomy if vascular access is an issue. Stamm Gastrostomy in premature infants is known to be prone to complications due to the extremely delicate skin that may be affected severely by leakage of gastric contents and breakdown of the gastrostomy site; however, a Stamm gastrostomy as a temporary measure could be life-saving, until it is found to be safe to reinsert the orogastric or nasogastric tube. Percutaneous devices such as percutaneous endoscopic gastrostomy (PEG) or PEG button are unsuitable for use in this age group. The time for the esophagus to heal and to attempt a new tube insertion remains unclear and there is no consensus on this issue. The safest approach would be to perform an upper gastrointestinal oral contrast study anytime 7–10 days after the perforation, and if no leak is observed the placement of a new tube could be attempted. After placement of the new tube, it is mandatory to perform a film with or without a contrast study through the tube to ensure correct placement before commencement of feeds.

The mainstay of non-operative management of removing the misplaced tube, making the patient nil by mouth, commencing IV antibiotics and total parenteral nutrition are agreed upon in the literature [6,10,11]. Karabulut et al. [28] reported using octreotide in the pediatric population which appeared to reduce length of stay in patients with esophageal injury, but there is no evidence for its use in NEPs. Likewise, Rollins et al. [29] had successful outcomes with the use of covered esophageal stents in children; again, there is no evidence for their use in NEPs. Preventative measures, including gently warming the catheter to soften it, may reduce the incidence of NEP [30]. In two of our patients, drainage of the pleural cavity was necessary, again echoing the literature, where this was required in cases of tension pneumothorax [1], simple pneumothorax [5], pneumothorax with pleural effusion [10] or pleural effusion alone [21]. Not seen in our cohort, there may also be pneumoperitoneum requiring peritoneal drain placement [11]. It is important to note that whilst the NEP was correctly identified in all of our patients, NEP may be misdiagnosed as esophageal atresia or other congenital abnormality [11], which may lead to a thoracotomy, where the true diagnosis is confirmed [6,17,31]. None of our patients required surgery to repair the perforation, though one underwent gastrostomy insertion allowing earlier enteric feeds, which is also reported in the literature [17,28]. The literature suggests surgical intervention may also be required as part of sepsis management, for example in cases of mediastinal abscess [6]. Contrast radiography may have a place in ensuring that feeds are not started when there is an on-going leak. The added benefit of this imaging technique is the ability to insert an enterogastric tube under fluoroscopic guidance, allowing enteral feeds to be started and bypassing the leak This method was used in one of our patients, thereby avoiding the potential added morbidity from a gastrostomy insertion. Ultrathin flexible endoscopy [32] can also provide confirmation of no ongoing leak and allow a new feeding tube to be inserted under direct vision. However, limited access to ultrathin flexible endoscopes and lack of training to use them is likely to limit their use. 

In our small cohort, one patient died of the sequelae of prematurity. This is similar to Onwuka et al. [1] series of 24 patients, the largest in recent literature, which reported a mortality rate of 16%, due to complications of prematurity rather than the iatrogenic injury. Likewise, Thanhaeuser et al. [4] found a mortality rate of 28.6% in their series of extremely low birth weight neonates with upper gastrointestinal perforations, also secondary to complications of prematurity.

Although non-surgical management is advocated in most of the neonates, surgical interventions must not be ruled out in severe cases. The decision to intervene will always be difficult and should involve the neonatal intensive care team, the pediatric surgeons and the anesthesiologists to weigh the outcomes if a procedure is offered. The most difficult aspect of neonatal esophageal perforations is offering information to parents who are anxious after being informed about the incident. With outcomes of neonatal esophageal perforations based on various factors, it can be challenging to inform the approach as there are no standardized protocols to base the management plan on. The access to online information by the parents further adds to their confusion not only with regard to the interventions, but also with regard to the medical management with the administration of antibiotics and steroids. Premature infants with minor and major co-morbidities will be even more challenging to manage and making parents understand the approach to management is often demanding for the teams involved. This aspect has not been touched on in the literature and will need to be addressed; however, this will require a larger cohort of patients to offer figures of success or failure as part of such discussions.

Despite a 5-year data collection period and four European centers contributing, our sample size was small. This likely reflects the rarity of the condition, but limits the conclusions that can be reached, such as optimal length of treatment with antibiotics or nil by mouth time. The retrospective nature of the study relied on the completeness of historic records and their interpretation by the researchers at each center. The use of a protocolized data collection form goes some-way to mitigate this. Taking into account the small sample size and retrospective review, this paper adds to the body of evidence that conservative management of NEP is safe and effective. Further research into the optimal course length of antibiotics, how to safely balance commencing oral feeds vs the complications of prolonged TPN, and whether fluoroscopic investigation should be undertaken prior to commencing oral feeds is required.

## 5. Conclusions

NEP is a rare complication in a neonatal intensive care unit, but one with significant morbidity and mortality. Sudden deterioration after enterogastric tube insertion should raise the suspicion of NEP, with the diagnosis confirmed by chest and abdominal X-ray. A lateral X-ray may also be useful. There is consensus across the recent literature that a course of IV antibiotics, and total parenteral nutrition, with a time of nil by mouth is the mainstay of treatment. A chest drain may also be necessary. Surgical management may also be required, particularly if there is a persistent leak, or if there is evidence of abscess formation and sepsis. 

Preventative measures including awareness, softening enterogastric tubes and ensuring the position of the tube is confirmed prior to commencing feeds, should reduce the incidence of, and the morbidity related to, NEPs. For example, NEP should always be considered in the event of a ‘spontaneous’ pneumothorax where there has been a history of enterogastric tube insertion or change. 

## Figures and Tables

**Table 1:** Summary of patient demographics and management.

Demographics	Value
Number of cases (*n*)	8
Male gender, *n* (%)	7 (87.5)
Median gestational age at birth, weeks (range)	26 + 4 (23 + 4–39)
Median birth weight, g (range)	636 (511–3500)
Conventional ventilation, *n* (%)	5 (62.5)
High frequency oscillation ventilation, *n* (%)	2 (25)
**Circumstances around perforation**	
First naso-/oro-gastric tube insertion, *n* (%)	1 (12.5)
Second naso-/oro-gastric tube insertion, *n* (%)	5 (62.5)
After multiple naso-/oro-gastric tube insertions, *n* (%)	2 (25)
**Symptoms and diagnosis**	
Respiratory distress alone, *n* (%)	4 (62.5)
Respiratory distress + sepsis, *n* (%)	2 (12.5)
Asymptomatic-post-insertion chest X-ray, *n* (%)	2 (25)
**Management**	
No thoracotomy, *n* (%)	8 (100)
Chest drain insertion, *n* (%)	2 (25)
Gastrostomy insertion, *n* (%)	1 (12.5)
Days of antibiotic, median	10
Days to oral feeds, median	10
Mortality, *n* (%)	1 (12.5)

## Data Availability

Data is available on request.

## Supplementary Materials

The following supporting information can be downloaded at: https://www.mdpi.com/article/10.3390/children10020217/s1. File S1: Neonatal esophageal perforations (nep) study protocol.
