# Supplementary material for: Iatrogenic Neonatal Esophageal Perforation: A European Multicentre Review on Management and Outcomes
Source: Children (Basel). 2023 Jan 26;10(2):217. doi: 10.3390/children10020217 (PMC9955351; doi:10.3390/children10020217)
Supplement: Supplementary file 1 [file children-10-00217-s001.zip › children-2158635-supplementary.pdf]

January 2014- December 2018 (5 year survey)

Please fill one form per patient. Insert Patient ID** number for London as L1, L2, L3 ….

Participant (Center)		
*Patient ID***		
Gender (M/F)		
Gestational age at birth(weeks)		
Weight of patient at birth (grams)		

Associated Anomalies	*Syndromic patient (Y/N)(If Y- specify)*	
	Cardiac anomalies (Y/N)(If Y- specify)	
	Musculoskeletal anomalies(Y/N)(If Y- specify)	
	Gastrointestinal anomalies(Y/N)(If Y- specify)	
	Urogenital anomalies (Y/N)(If Y- specify)	
	Others (Y/N)(If Y- specify)	
Perforations	*Days after birth when perforation occurred*	
Respiratory Status (pre - perforation)	*Normal breathing (Y/N)*	
	Ventilated (Y/N)	
	High frequency oscillated ventilation (Y/N)	
	ECMO (Y/N)	
	Others (please specify)	
Perforation & Nasogastric Tube	*OG/NG tube placed immediately after birth (Y/N)*	
	Occurred during first NG tube placement (Y/N)	
	Occurred during first NG tube change (Y/N)	
	After multiple NG tube changes (Y/N)	
	Size of NG tube (if known)	
Perforation during anesthesia	*Did perforation occur during anesthesia induction* – *Yes*	
	No	
Site of perforation (if known)	*Proximal esophagus (Y/N)*	
	Mid esophagus (Y/N)	
	Distal esophagus (Y/N)	
Establishment of Clinical Diagnosis	*Recognized during OG/NG tube insertion (Y/N)*	
	Asphyxia after pneumo-mediastinum (Y/N)	
	Respiratory distress (Y/N))	
	Pulmonary infections (Y/N)	
	Onset of sepsis/shock (Y/N)	
	Other (please specify)	
Conservative Management	*Was an OG/NG tube reinserted (Y/N)*	
	- *Ultrasound guidance (Y/N)*	
	- *Fluoroscopic guidance(Y/N)*	
	- *Endoscopic guidance (Y/N)*	
	Chest tube required (Y/N)	
	- *Duration of chest tube (Y/N)*	
	Total parenteral nutrition (Y/N)	
	- *Duration of TPN*	
	- *Days until start of oral feeds*	
	- *Days until full oral feeds*	

Medications	*Steroids (Y/N)*	
	- *If yes specify*	
	- *If yes duration*	
	Antibiotics (Y/N)	
	- *If yes specify*	
	- *If yes duration*	
	H2 blockers (Y/N)	
	- *If yes specify*	
	- *If yes duration*	
	Other medications (Y/N)	
	- *If yes specify*	
	- *If yes duration*	

Surgery	*Was surgery offered (Y/N)*	
	- *If yes specify procedure (e.g. Nitonol stent / Gastrostomy/ Jejunostomy etc.)*	

Morbidity	*Esophageal stenosis/stricture (Y/N)*	
	Pleural effusions (Y/N)	
	Mediastinal abscess formation (Y/N)	
	Emphysema (Y/N)	
	Pericardial effusions (Y/N)	

Outcomes	*Survival with minimal morbidity (Y/N)*	
	Survival with severe morbidity (Y/N)	
	- *If yes specify*	
	Asphyxia with severe neurology (Y/N)	

Mortality (If yes, please answer this section)	*Days from perforation to lethality*	
	Possible cause of lethal outcome:	
	- *Sepsis*	
	- *Asphyxia*	
	- *Cardiac reasons*	
	- *Others (please specify)*	
